# A retrospective assessment of metropolitan religious adherence rate, individual and neighborhood social capital and their impact on women’s health

**DOI:** 10.1186/s12889-019-7530-6

**Published:** 2019-08-28

**Authors:** Kim Nichols Dauner, Neil A. Wilmot

**Affiliations:** 1 Health Care Management Program, Department of Economics, University of Minnesota Duluth, 1318 Kirby Drive, Duluth, MN 55812 USA; 2 Department of Economics, University of Minnesota Duluth, 1318 Kirby Drive, Duluth, MN 55812 USA

**Keywords:** Social capital, Religion, Community level, Health, Women, Fragile families

## Abstract

**Background:**

Social capital is a multilevel construct impacting health. Community level social capital, beyond the neighborhood, has received relatively less attention. Moreover, the measurement of community level social capital has tended to make use of aggregated individual data, rather than observable community characteristics.

**Methods:**

Herein, metropolitan religious adherence, as an observable community-level measure of social capital, is used. We match it to city of residence for 2826 women in the Fragile Families Childhood Wellbeing Study (a cohort study) who have lived continuously in that city during a nine-year period. Using ordered logistic regression with clustered standard errors to account for area effects, we look at the relationship between metropolitan religious adherence and self-rated health, while controlling for lagged individual, neighborhood, and socioeconomic factors, as well as individual level religious attendance.

**Results:**

Religious adherence at the community level is positive and statistically significant; every 1% increase in area religiosity corresponds to a 1.2% increase in the odds of good health.

**Conclusions:**

These findings shed light on a possible pathway by which social capital may improve health, perhaps acting as a stress buffer or through spillover effects of reciprocity generated by exposure to religion.

## Background

Social capital is a multilevel construct composed of both individual (social networks, participation in social and civic organizations) and contextual community factors (social cohesion of neighborhoods, workplaces, institutions; the economic environment; organizations available to address social welfare; funding levels), that, when taken together can improve the efficiency of society by facilitating coordinated action [1]. It is recognized that social capital is an important health determinant [2, 3].

Within the public health literature, social capital is frequently conceptualized as a subset of social cohesion, a larger concept referring to the closeness and solidarity within groups, and measured through an individual’s or a community’s sense of belonging, trust and norms of reciprocity [4]. Social capital generates social cohesion, and research at the individual level indicates that increased social capital, defined as generalized trust in others, social participation, and reciprocity, is positively associated with lower mortality [2, 5], lower odds of depression [6], and improved self-rated health [7–9]. These relationships between social capital and health have also been identified within the sample of mothers participating in the Fragile Families and Childhood Wellbeing (FFCWB) study [6, 9], which has followed a cohort of children and their families born in large U.S. cities to mostly minority, unmarried parents and who tend to be at greater risk of falling into poverty [10].

While neighborhood physical and social environment seems to have a positive impact on some health outcomes including coronary heart disease [11] and mortality [12], a recent assessment of the literature found that observational designs and multilevel models tended to show small, statistically significant effects of the neighborhood but point out that, while rare, studies that use experimental methodologies tend to find more mixed results [13]. The reality is people live in neighborhoods embedded in larger units (e.g. cities, states, countries) and participate in institutions (workplaces, religious, educational) within these larger communities. Synergies occur between people and place [14]. As such, it is not surprising that many have called for measures at multiple levels and capturing structural and collective social function attributes [5, 15–17].

Much of the current multilevel research on social capital and health have used aggregated survey data from population-based studies to make inferences on the relationship between individual and higher order social capital. These studies suggest that the social capital available within a person’s community or workplace impacts health, independent of individual social capital and this impact is positive regardless of setting, study design or health outcome measured [13]. Moreover, these hold up using various constructions of community beyond a neighborhood [18, 19]. While aggregated survey-based responses are important as communities are made up of people, they are still limited, based on perceptions, which may or may not match behavior [20].

Putman and Coleman in their original conceptualizations of social capital, posited that religious involvement is a social capital generator [21, 22]. Religious social capital is multidimensional and encompasses structural components of the religious institutions available in a community as well as the values and norms, trusting relationships and social support derived through those participating in those institutions [23]. Religion influences the formation of individual and societal values, shapes viewpoints on human behavior, promotes norms on the treatment of others, and increases social connection [24, 25]. While religion can create a sense of cohesion between individuals and social groups, it can also exert social control amongst both individuals and groups [26]. Religious attendance has been shown to increase social trust, more so than religious membership [24]. Further, religious involvement has been shown to improve health perception [27], delay pulmonary function decline [28], and lower mortality [29]. Among women, both religious activity and spiritual experiences are independently associated with health [27]. Membership in a religious congregation, even without participation, is associated with increased likelihood of status-bridging social capital through friendships [30]. Further, these relationships may be even more impactful in disadvantaged populations [31].

There is evidence of a relationship between religious organization at the community level and public health outcomes. A higher number of religious organizations per capita has been associated with lower likelihood of alcohol, drug, and mental health disorders [32]. At the county level, Beyerlein and Hipp found an association between lower crime and affiliation with mainstream Protestant and Catholic religions [33]. Blanchard and colleagues also find evidence of lower county level mortality in counties with more mainline Protestant and Catholic congregations, though counties with more conservative Protestant congregations had higher mortality [34]. Maselko and colleagues posit religious social capital as a strong population health determinant reflecting the fact that religious communities are formalized groups of people with similar beliefs and backgrounds, and that this religious social capital may substitute for low neighborhood social capital when it comes to health [23].

In the present study, we expand upon the current literature by incorporating an observable measure of religious social capital acting at the community level. We investigate this relationship among women across 20 different metropolitan areas of the U.S. participating in the FFCWB study by matching metropolitan religious adherence rate to the women’s city of residence. We restrict our analysis to the women who have not moved within the nine-year time frame and we use lagged measures of social capital to decrease endogeneity concerns. This study offers several contributions to the social capital and health literature and complements research using aggregate measures of social trust / capital. First, we use an observable, behavior-based measure of social capital at the community level. Second, we control for individual and neighborhood social capital. Third, our use of metropolitan religious adherence captures the multiple dimensions or religious social capital (structural components, values and norms, trusting relationships and social support) and suggests religious participation.

## Methods

### Data

The FFCWB study follows a cohort of about 5000 children born to low-income parents in 20 major U.S. cities (in 15 states), during the years 1998–2000. By design, approximately three-quarters of the mothers were unmarried. Face-to-face interviews were conducted with 4898 mothers shortly after giving birth. Both parents were interviewed at the time of their infant’s birth and then again one, three, five, and 9 years later. The study was designed to provide information on the conditions and capabilities of new parents, the determinants and trajectories of parental relationships, and the consequences for parents and their children of health, child welfare, and social service policies as well as other aspects of their environment [10]. We used data obtained from both the publically available FFCWB database, through Princeton University’s Office of Population Research, as well as Fragile Families Census Tract Measures and Fragile Families Labor Market and Macroeconomic Data, both of which are part of their restricted use files. The Institutional Review Board at the University of Minnesota approved the study’s methods and subsequent use of the restricted data. Our study examines data only from the mothers, as there was a disproportionate response rate for the fathers beyond the initial wave of the FFCWB study. Since our main variable of interest is community level religious adherence, the sample was further limited to mothers who had not moved over the first nine-years of the study (72.5% of women). This research builds off our prior work looking solely at individual level variables of social capital within the population of FFCWB study mother and which has been previously published [9].

### Dependent variable – self-rated health

The dependent variable, self-rated health (*SRH*), was obtained from the nine-year wave of the survey (2007 to 2009). Self-reported health has been shown to be a valid measure of general health and its use is consistent across studies examining the relationship between social capital and health [35]. Mothers were asked to rate their overall general health on a five-point scale ranging from excellent (= 5) to poor (=1).

### Individual measures of social capital

We included individual level religious participation as a dichotomous variable with those responding that they attended religious services once a week or more frequently being characterized as participators. Based on individual’s responses to questions from the five-year follow up survey of mothers (2003 to 2006), we also created three indices of social capital, informed by the 2006 Social Capital Community Survey [36] and our prior work [9]. Table 1 lists the specific questions used. The first index, s*ocial support and trust*, was constructed from several dichotomous questions (yes/no), meant to gauge whether mothers had access to emotional and tangible supports when needed. The constructed social capital measure, is a summation of the “yes” (= 1) responses to each question.

**Table 1:** Individual Level Social Capital Indexes

1. Social Support and Trust index	
Do you have someone you could trust to look after your child if you are away?	
Do you have someone who would loan you $200?	
Do you have someone who would provide you a place to live?	
Do you have someone who would provide you with emergency childcare?	
Do you have someone who would co-sign for a bank loan ($1000)?	
Do you have someone you could share confidence with?	
2. Perceived Neighborhood Social Cohesion	
*What level of agreement do you have with the following statements?*	
You are willing to help a fellow neighbor.	
The neighborhood is viewed as a close-knit community	
People in this neighborhood generally get along with each other.	
People in this neighborhood general share the same values.	
Gangs are a problem in the neighborhood (reverse coded)	
3. Perceived Neighborhood Social Control	
Mothers were asked about the likelihood of neighbors to intervene if children are	
Skipping school	
Spray painting a building	
Showing disrespect to an adult	
Likelihood of a neighbor intervening to diffuse a fight	
Willingness to intervene to save a local firehouse	

The *perception of neighborhood social cohesion* scale was constructed using the respondent’s level of agreement to five Likert-type questions, using validated measure of social cohesion within the FFCWB [37]. The question regarding gangs being a problem was recoded so that lower levels of agreement were coded as being more positive. After recoding, the mean response to the Likert-type questions expressing level of agreement was used to construct the index, with higher scores indicating higher levels of neighborhood social cohesion. The *perceived neighborhood social control* index uses a validated measure within the collective efficacy construct [37] and is constructed from five Likert-type scale questions. The individual’s mean response was used to construct the index, with higher scores indicating higher levels of perceived neighborhood social control. Internal consistency for both scales is reasonable; Cronbach’s alpha for the perception of neighborhood social cohesion is 0.75 and is 0.88 for the perceived neighborhood social control scale. While the three constructed measures of social capital are related, the pairwise correlation values are low; the highest degree of correlation (0.465) is found between perceived neighborhood social cohesion and perceived neighborhood social control, while the remaining correlations are below 0.23.

### Community level social capital

The religious adherence rate of the metropolitan area in which the woman resides was used as a proxy for community level social capital (*Religious Adherence*). Data on the Adherence Rate of Across Religions, in 2000, comes from adherent totals collected by the Association of Statisticians of American Religious Bodies (ASARB) and was obtained from Social Explorer (RCMS:T2) for each of 18 metropolitan areas represented in the FFCWB (while the FFCWB women live in 20 different cities, those cities represent 18 distinct metropolitan areas) [38]. We used the adjusted totals to account for religious adherence in historically African-American denominations which are under-represented in the ASARB data.

### Explanatory variables

At the individual level, we controlled for a number of socioeconomic, demographic, and behavioral variables known to influence self-rated health. All measures were lagged and obtained from the five-year follow-up interview (t – 1), with the exception of education which comes from the baseline interview. These included level of education, age, race, income, relationship status, employment status, poverty, smoking behavior, and SRH. Education was measured as highest educational level obtained. Race is categorized by the FFCWB as “black” “Hispanic” “white” or “other.” Income was categorized into several mutually exclusive categories, including less than $30,000, $30,000–$59,999, and $60,000 and above, while a fourth category for “not reported” was included. The mothers’ relationship status was measured as two separate binary variables, the first related to whether she is married and the second based on cohabitation. The inclusion of multiple measures of relationship status is an attempt to capture the diversity of relationship types among women in the sample. Employment status was a binary measure “yes” or “no.” Poverty status was a binary measure, with “yes” indicating that the individual had received welfare or food stamps in the previous 12 months (at time t – 1). Whether the person has smoked within the last 30 days was a binary “yes” or “no” measure (at time t – 1). Also, previously reported health, obtained from the previous FFCWB wave, *SRH*_*t* − 1_, was dichotomized into ‘favorable’ health (excellent, very good, good) and ‘unfavorable’ (fair, poor) health.

We also controlled for community level socioeconomic factors. The unemployment rate (*Unemployment*), measured as the percentage of the civilian labor force (16+) unemployed in the census tract where mother lived at the time of the baseline interview (conducted between 1998 and 2000), was obtained from the geo-coded Fragile Families data set, which is derived from 2000 census data. In addition, we controlled for neighborhood disadvantage, given its effect on health and wellbeing [39], particularly among poorer families [40]. Similar to previous studies, we used principle component analysis to generate a composite score of neighborhood disadvantage based on 6 U.S. census tract characteristics [39, 40]. The full set of indicators used to create the neighborhood disadvantage index is presented in Table 2 and includes measures of unemployment, welfare, poverty, high school education, college degree (reverse coded), and the percentage of female head of households in a given census tract. Each of the individual indicators had factor loadings greater than 0.70, so all six were retained in the analysis. The results of the principle component analysis suggested the presence of one primary factor, which could be described as “neighborhood disadvantage” [39]. Then, standardized Z-scores for each of the six components were summed, with higher scores indicating higher levels of disadvantage. The neighborhood disadvantage index for the census tracts in our sample ranges from − 7.90 to 16.73. A binary variable was created based on the median value (− 0.54), such that those neighborhoods with scores above the median received a value of 1 (“more disadvantaged”), and neighborhoods with scores below the median, received a value of 0. Finally, we note a small negative correlation between the disadvantage index, just described, and the neighborhood social capital measures of less than − 0.20.

**Table 2:** Factor Pattern of Neighborhood Indicators

Census Tract Indicator	Factor Loading
% Persons age 16 years & older in Labor force	0.706
% Households with public assistance	0.867
% Families below the poverty level	0.909
% (Females) population that have not graduated high school	0.841
% Female Head of household (with children under 18 present)	0.818
% (female) population with associates degree or higher (reverse coded)	0.702

### Analysis

We account for the temporal ordering using lagged measures of social capital (t-1), relative to the dependent variable. In particular, we tested the hypothesis that metropolitan religious adherence at time (t – 1) is positively associated with SRH at time (t), independent of individual level social capital and neighborhood disadvantage. To examine these hypotheses, an ordered logistic regression model is estimated, given the intrinsic ranking within the dependent variable. To account for the clustering of observations within metropolitan areas, the estimation utilized clustered standard errors, which allows for the observations on individuals in the same community to be correlated and is appropriate since we are interested in the general contribution of the higher order variable (religious adherence) on a given outcome, rather than the ranking of metropolitan areas relative to each other [41]. Let *i* denote the *i*^th^ of *N* individuals in the sample and *g* denote the *g*^th^ of *G* clusters, then equation for the logistic model is given as

1 $$ Logit\left({H}_{i,g}\right)=\beta X+\varphi {S}_{i,g}+\gamma {C}_g+{\varepsilon}_{i,g} $$

*H* is *SRH* in period *t*, *X* includes socio-economic and demographic variables in period *t-1*. Individual-level measures of social capital are given in *S*, at time *t – 1*, while *C* represents the community-level variables (year 2000). A key assumption is that observations are independent across groups, but not necessarily within groups. This implies the errors are uncorrelated across clusters while errors within clusters may be correlated,

2 $$ E\left[{\varepsilon}_{i,g}{\varepsilon}_{i,g\hbox{'}}|{\mathbf{x}}_{i,g}{\mathbf{x}}_{i.g\hbox{'}}\right]=0\kern0.75em \mathrm{unless}\kern0.5em g=g\hbox{'}. $$

Given error independence across clusters, the variance – covariance matrix, Ω_*g*_, has a block- diagonal structure.

The hypotheses of interest in the current study are represented by *φ,* which is expected to be positive, while the community level measures, represented in *γ*, are expected to vary with each measure. Each dimension of social capital was included individually in a model examining its impact on SRH, while controlling for the covariates (models 1–4), while a fifth model, which included all measures of social capital, was also examined. The last two also include an interaction term between religious adherence and religious participation, as others have reported a significant interactive effect [42]. These analyses build upon our prior work [9] with the inclusion of the community level religious adherence measure and socioeconomic variables. To examine the robustness of the estimates a logistic regression model using a dichotomous measure of SRH (excellent, very good, and good vs. fair and poor health) was estimated. The logistic regression was also used to calculate marginal effects. For brevity, the results of the logistic regression are not presented, but are available from the authors upon request.

## Results

The final sample used in the analysis included 2826 women (of the original 4898 mothers participating in the baseline FFCWB interview) who participated in the five and nine-year FFCWB surveys and who remained in the same metropolitan area as baseline. It should be noted that the sample sizes of several models have fewer observations due to non-response to interview questions. Frequencies for the individual social capital measures and explanatory variables are presented in Table 3. We note the majority of the sample consists of those who self-reported favorable health (‘excellent’, ‘very good’, ‘good’). The average age of the sample is approximately 34 years old, and nearly one in three mothers is married. Of those who reported annual income, 35.5% are earning less than $30,000 per year, the median income calculated from the sample, while approximately 40% have received income from welfare / TANF or from food stamps, in the previous 12 months. The religious adherence rate of the 20 metropolitan areas is, on average, nearly 62%, ranging between 45.8% (San Jose) and 78.8% (New York). The average level of census tract unemployment across the sample is 0.1094. Nearly half of those with favorable health reside in a disadvantaged neighborhood, while over 60% of those with unfavorable health reside in a disadvantaged neighborhood. As one might expect, the correlation between the disadvantage index and the unemployment rate is elevated (0.7139), while the correlation between the measures of social capital and community level variables appears low.

**Table 3:** Frequency of Variables Stratified by Self-Reported Health

			Health Status			
			Favorable		Unfavorable	
	Freq.	Mean	Freq.	Mean	Freq.	Mean
Total	2826		82.7%		17.3%	
*Social Capital Measures*						
*Community Level*						
Religious Adherence	100.0%	61.76	82.7%	61.96	17.3%	60.82
*Individual Level*						
Social Support and Trust	93.6%	4.77	93.7%	4.88	92.9%	4.24
Perceived Neighborhood Social Control	92.3%	3.23	92.5%	3.26	91.2%	3.11
Perceived Neighborhood Social Cohesion	92.8%	2.98	93.0%	3.01	92.0%	2.80
Religious Participation	93.2%		36.0%		14.5%	
Explanatory Variables						
*Community Level*						
Unemployment	100.0%	0.10	82.7%	0.106	17.3%	0.124
Disadvantage Index	100.0%	0.50	82.7%	0.479	17.3%	0.602
*Individual Level*						
Health _*t-1*_	80.5%		71.0%		9.5%	
Nonsmoker	64.9%		55.7%		9.1%	
Age	100.0%	34.3	82.7%	34.37	17.3%	34.13
Employed	56.7%		49.4%		7.3%	
Married	30.3%		24.9%		3.7%	
Cohabitating	55.1%		46.2%		8.9%	
Race						
White	19.3%		16.9%		2.4%	
Black	52.4%		43.0%		9.4%	
Hispanic	24.9%		20.0%		5.0%	
Other	3.1%		2.7%		0.5%	
Poverty	40.4%		30.8%		9.6%	
Income						
Less than $30,000	35.5%		27.9%		7.6%	
$30,000 - $59,999	22.3%		19.7%		2.6%	
$60,000 +	12.3%		11.5%		0.7%	
Missing Income	29.9%		23.5%		6.4%	
Education						
Less than High School	39.8%		30.7%		9.1%	
High School Graduate	26.6%		22.0%		4.6%	
Some College	23.7%		20.6%		3.1%	
College Graduate	9.7%		9.3%		0.5%	

Note: Favorable health is considered a Self-Reported health value of 5 (Excellent; *n* = 589), 4 (Very Good; 868) or 3 (Good; 879), while Unfavorable is comprised of a SRH outcome of 2 (Fair; 427) and 1(Poor; 60). Poverty is defined as having received welfare or food stamps within the last 12 months. The mean is provide for the measures of social capital, Religion rate, Unemployment rate and age

The estimated odds ratios of the ordered logistic regression models investigating the relationship between SRH and four measures of social capital are presented in Table 4. At the community level the metropolitan religious adherence rate is positive and highly statistically significant (Table 4), across all models. These results indicate that a 1% increase in the religiosity of the metropolitan area leads to an increase in the odds of reporting good health by at least 1.2%. At the individual level, women with higher levels of social support and trust at time *t - 1*, rate themselves to be healthier at time *t*, as indicated by the positive and statistically significant odds ratio (Models 1 & 5). The results for *social support and trust* indicate that a unit increase in the social support and trust measure leads to an increase in the odds of reporting good health by approximately 10%. The perceived neighborhood social control (Model 2) and perceived neighborhood social cohesion (Model 3) variables are statistical significance at the 1% level. However, in the full model (Model 5), perceived neighborhood social control is no longer significant. The odds ratio for individual religious participation are significant and greater than 1, shown in both Models 4 and 5, indicating an increase in the odds of reporting favorable health if the individual is religious. However, the interaction term, also significant in these two models, indicates that a religious individual located in a highly religious community, is less likely to report favorable health. Noticeably, the inclusion of both religious participation and the interaction term does not change the direction nor the significance of the community level religious adherence variable.

**Table 4:** Ordered Logit Estimation of Social Capital Measures on Self-Reported Health

	***n =***	Model 1	Model 2	Model 3	Model 4	Model 5
		2629	2594	2610	2605	2584
Indices of Social Capital						
*Community Level*						
Religious Adherence		1.012 *** (0.003)	1.012 *** (0.003)	1.013 *** (0.004)	1.02 *** (0.005)	1.021 *** (0.005)
*Individual Level*						
Social Support and Trust		1.103 *** (0.033)	..	..	..	1.083 *** (0.032)
Perceived Neighborhood		..	1.155 *** (0.054)	..	..	1.067 (0.049)
Social Control						
Perceived Neighborhood		..	..	1.329 *** (0.115)	..	1.251 ** (0.119)
Social Cohesion						
Religious Participation		..	..	..	3.319 *** (1.412)	3.637 *** (1.402)
*Interaction*						
Religion interaction		..	..	..	0.979 *** (0.007)	0.978 *** (0.007)
Explanatory Variables						
*Community Level*						
Unemployment Rate		0.753 (0.664)	0.639 (0.554)	0.817 (0.692)	0.795 (0.674)	0.694 (0.604)
Disadvantage Index		0.876 (0.101)	0.882 (0.100)	0.883 (0.100)	0.853 (0.092)	0.898 (0.101)
*Individual Level*						
Health _*t-1*_		5.799 *** (0.880)	5.975 *** (0.946)	5.773 *** (0.892)	6.133 *** (0.950)	5.722 *** (0.911)
Nonsmoker		1.484 *** (0.148)	1.497 *** (0.148)	1.465 *** (0.143)	1.479 *** (0.145)	1.491 *** (0.140)
Age		0.981 *** (0.007)	0.977 *** (0.006)	0.976 *** (0.006)	0.980 *** (0.006)	0.979 *** (0.006)
Employed		1.101 (0.072)	1.107 (0.071)	1.109 (0.071)	1.109 (0.067)	1.09 (0.073)
Married		0.933 (0.111)	0.978 (0.121)	0.964 (0.116)	0.954 (0.115)	0.972 (0.120)
Cohabitating		0.955 (0.081)	0.971 (0.088)	0.977 (0.084)	0.969 (0.085)	0.951 (0.080)
Race						
Black		0.986 (0.117)	1.018 (0.127)	1.001 (0.122)	0.986 (0.117)	1.052 (0.134)
Hispanic		0.852 (0.103)	0.866 (0.110)	0.857 (0.103)	0.854 (0.106)	0.905 (0.117)
Other		1.040 (0.255)	1.098 (0.287)	1.050 (0.251)	1.009 (0.247)	1.097 (0.281)
Poverty		0.822 ** (0.075)	0.810 * (0.077)	0.816 ** (0.077)	0.804 ** (0.075)	0.829 * (0.080)
Income						
$30,000 - $59,999		1.075 (0.083)	1.084 (0.081)	1.075 (0.076)	1.111 (0.081)	1.05 (0.079)
$60,000 +		1.332 * (0.208)	1.337 * (0.219)	1.302 * (0.207)	1.386 * (0.217)	1.258 (0.213)
Missing Income		0.930 (0.075)	0.939 (0.076)	0.904 (0.071)	0.928 (0.080)	0.931 (0.080)
Education						
High school graduate		1.059 (0.099)	1.060 (0.100)	1.062 (0.101)	1.091 (0.105)	1.032 (0.095)
Some College		1.072 (0.096)	1.080 (0.097)	1.078 (0.101)	1.109 (0.101)	1.043 (0.094)
College Graduate		1.846 *** (0.292)	1.897 *** (0.313)	1.874 *** (0.303)	1.978 *** (0.325)	1.79 *** (0.284)
Pseudo R^2^		0.064	0.0632	0.0647	0.066	0.068

Note: The Dependent variable, Self-Reported health, ranges from excellent health (=5) to poor health (=1). The size of the sample, n, is provided. Clustered Robust standard errors are provided in parentheses. * indicates significance at the 10% level, ** indicates significance at the 5% level while *** indicates significance at the 1% level. Individual level religion is a binary variable

In contrast, neither the unemployment rate nor the disadvantage index appears to be significant in explaining SRH. Previously reported level of health is a positive and statistically significant predictor of current self-reported health, while being a nonsmoker at time (t – 1), is associated with favorable SRH (t). The individual’s age, at time (t – 1) was negatively and statistically significant predictor of favorable SRH in period (t),. Finally, the level of education attained by the mother at the time of the child’s birth (baseline wave of the FFCWB) appears positively and significantly associated with SRH; being a college graduate nearly doubles one’s odds of reporting favorable health.

For ease of exposition the marginal effects from the logistic regression model (where SRH was dichotomized) are presented in Table 5. The results suggest that a unit increase in the metropolitan’s religious adherence rate increases the probability of favorable SRH by approximately 0.14%, across the three models. In addition, a unit increase in an individual’s level of social support increases the probability of reporting favorable health status by 0.9%. The increases in the probability of reporting favorable health status observed for perceived neighborhood social control is approximately 1%, while the probability for perceived neighborhood social cohesion is much higher at 2.0%, though the latter is not a statistically significant effect. In contrast to the effect of community level religious adherence, individual religious participation leads to an approximately 1% increase in the probability of reporting favorable health. The marginal effect of the interaction term, while negative, is not statistically significant. In the full model (Model 5), the marginal effects are similar, though muted, with the effect of perceived neighborhood social control no longer statistically significant.

**Table 5:** Marginal Effects from Logit Estimation of Social Capital Measures on Self-Reported Health

	Model 1	Model 2	Model 3	Model 4	Model 5
	(s.e.)	(s.e.)	(s.e.)	(s.e.)	(s.e.)
Social Capital Measures					
Community Level					
Religious Adherence	0.0014 *** (0.0004)	0.0015 *** (0.0005)	0.0014 *** (0.0004)	0.0014 *** (0.0004)	0.0015 *** (0.0004)
Individual Level					
Social Support and Trust	0.0095 ** (0.0042)	..	..	..	0.0071 * (0.0038)
Perceived Neighborhood Social Control	..	0.0106 * (0.0064)	..	..	0.0032 (0.0041)
Perceived Neighborhood Social Cohesion	..	..	0.0207 (0.0137)	..	0.0149 (0.0115)
Religious Participation	..	..	..	0.0095 (0.0647)	0.0201 (0.0524)
Interaction					
*Relg Adh.* x *Relg Part.*	..	..	..	−0.0003 (0.0010)	−0.0004 (0.0009)

Note The marginal effects, calculated at the mean, for the dichotomous dependent variable are presented. If the variable was dichotomous the median was used as an alternative to the mean. This includes the individual religion variable. The table also presents the standard error of the marginal effect, in the parentheses

Overall, the results indicate the importance of metropolitan religious adherence rate, and individual level social capital measures, the majority of which remain statistically significant in the final model (Table 4, Model 5). In contrast, onlyperceived neighborhood measures of social capital is no longer statistically significant. The results of a joint test of significance, at the 1% level, suggest the neighborhood measures should remain in the model. The marginal effect of social support and trust, along with religion, remains significant (Table 5, Model 5). Similar covariates are found to be significant in this broader model. The robustness of the results of the relationship between these measures of social capital and SRH are further investigated using a dichotomous logistic regression model, where SRH is based on the two categories, ‘favorable and unfavorable. The results of this logit model lend support to the findings presented here (results not presented in tables but are available from the authors).

## Discussion

Our findings fit in with prior findings on the association between an ecological measure of religious social capital and a health outcome [32, 33] and add to the diversity of documented social capital-health relationships [43]. Our findings expand upon previous findings of the relationship between social capital and health among FFCWB participants and point to the added contribution of religious social capital at the population level. Interestingly, prior research has found individual social participation, including individual religious participation, not significantly associated with health among women [9, 44]. At the same time, it cannot be ignored that religion can exert negative social capital [33]. Different compositions of religious denominations at the community level may impact health differentially [34]. Stroope and Baker found that in highly religious contexts, religious individuals were more likely to report poor health; however, in less religious contexts there were no differences based on individual religious attendance [42]. Similarly, we found a significant interaction between individual religious participation and community level religious participation in the FFCWB Study population. Other research has suggested there are spillover effects of exposure to religion, independent of individual religious attendance that are health promoting [23]. Further, our research sheds light on a possible pathway by which social capital may improve health, perhaps acting through a stress-buffering mechanism [32]. Finding a significant impact of religious adherence at the population level complements the positive impacts others have found of aggregated population level measures of social capital on health [19]. This study is one of only a few to investigate individual, neighborhood and ecological levels of social capital together using an observable, community-level measure of social capital.

There is a need for further research on the intersection of race, ethnicity, poverty, religious social capital and health. Irwin and colleagues found a more pronounced protective effect of social capital in disadvantaged populations [31]. Meanwhile, Rupasingha, Goetz, and Freshwater found a positive relationship between racial homogeneity and social capital production [45]. In contrast, Carpiano found both positive and negative effects of social capital among urban women caregivers [46]. Putnam found increased social capital production among mothers, though lower social capital among unmarried persons [47]. Mitchell and LaGory have mixed findings on the effect of social capital on health in high-poverty, high-minority, inner-city communities [48]. Liu, Austin, and Orey find a relationship between participation in black churches and voting behavior, suggesting black churches as places of social capital production [49]. Because the FFCWB focuses exclusively on women, most of whom are members of racial and ethnic minority groups, and who gave birth to children out of wedlock and are at greater risk for poverty, it is unclear how to contextualize our findings in light of the previous literature. Nevertheless, we found a positive effect of metropolitan area religious social capital even while controlling for the fact that most of the women in the FFCWB were living in neighborhoods with high levels of disadvantage. This seems to support the health supporting relationship between religion and health. Churches in general, and African-American churches in particular, are well-known for their ability to influence health behaviors [50].

The present study has limitations. First, we cannot rule out reverse causality. However, the fact we looked at lagged measures of social capital in reference to health and restricted our analysis to women who lived within the same city during that 10-year period of time is a strength. Second, we also don’t know how religious adherence may have changed over this time period. It is possible that cities had events that altered the religious adherence of the entire community and we were not able to capture that. At the same time, there is no research informing us as to what is the right amount of time required to adequately develop social capital and then have it impact health. Third, the use of self-rated health as an endpoint (as compared to mortality) may be a limitation. However, we feel self-rated health implies a perception of quality of life that mortality doesn’t. Last, it is possible that omitted variables, particularly those at the community level, impact our findings in some way. With social capital research continuing to move beyond the individual level, broader conceptualizations of social capital and their measurement across populations and place are an important focus for future research [4].

## Conclusions

Our findings contribute to our understanding of how religion impacts health at a community level and shed light on a possible pathway by which social capital may improve health, perhaps acting as a stress buffer or through spillover effects of reciprocity generated by exposure to religion. Recent research on social capital interventions call attention to the individualized focus of most interventions and the subsequent need to design community-, systems- and policy-focused social capital interventions and evaluate their effects on social capital and health outcomes [51, 52]. To this end, our findings point towards incorporating religious organizations as part of a health in all policies approach [53] or other systems level approach to addressing social determinants of health and promoting health equity.

## Data Availability

The datasets generated and/or analyzed during the current study are available from the Fragile Families and Childhood Wellbeing repository. Access to their public use data and information on obtaining the restricted use data are available through this link: https://fragilefamilies.princeton.edu/documentation

## Abbreviations

ASARB: Association of Statisticians of American Religious Bodies
FFCWB: Fragile Families Childhood Wellbeing Study
SRH: self-rated health

## References

[1] Putnam RD (1993). The prosperous community. Am Prospect.

[2] Kawachi I, Kennedy BP, Lochner K, Prothrow-Stith D (1997). Social capital, income inequality, and mortality. Am J Public Health.

[3] Portes A (1998). Social capital: its origins and applications in modern sociology. Annu Rev Sociol.

[4] Carrillo Álvarez E, Riera RJ (2017). Measuring social capital: further insights. Gac Sanit.

[5] Gilbert KL, Quinn SC, Goodman RM, Butler J, Wallace J (2013). A meta-analysis of social capital and health: a case for needed research. J Health Psychol.

[6] Wilmot NA, Dauner KN (2017). Examination of the influence of social capital on depression in fragile families. J Epidemiol Commun H.

[7] Giordano GN, Lindström M (2011). Social capital and change in psychological health over time. Soc Sci Med.

[8] Giordano GN, Björk J, Lindström M (2012). Social capital and self-rated health–a study of temporal (causal) relationships. Soc Sci Med.

[9] Dauner KN, Wilmot NA, Schultz JF (2015). Investigating the temporal relationship between individual-level social capital and health in fragile families. BMC Public Health.

[10] Reichman NE, Teitler JO, Garfinkel I, McLanahan SS (2001). Fragile families: sample and design. Child Youth Serv Rev.

[11] Diez-Roux AV, Javier Nieto F, Muntaner C, Tyroler HA, Comstock GW, Shahar E (1997). Neighborhood environments and coronary heart disease: a multilevel analysis. Am J Epidemiol.

[12] Yen IH, Kaplan GA (1999). Poverty area residence and changes in depression and perceived health status: evidence from the Alameda County study. Int J Epidemiol.

[13] Oakes JM, Andrade KE, Biyoow IM, Logan T (2015). Twenty years of neighborhood effect research: an assessment. Curr Epidemiol Rep.

[14] Arcaya MC, Tucker-Seeley RD, Kim R, Schnake-Mahl A, So M, Subramanian SV (2016). Research on neighborhood effects on health in the United States: a systematic review of study characteristics. Soc Sci Med.

[15] Meijer M, Röhl J, Bloomfield K, Grittner U (2012). Do neighborhoods affect individual mortality? A systematic review and meta-analysis of multilevel studies. Soc Sci Med.

[16] Macintyre S, Ellaway A, Cummins S (2002). Place effects on health: how can we conceptualise, operationalise and measure them?. Soc Sci Med.

[17] Lochner K, Kawachi I, Kennedy BP (1999). Social capital: a guide to its measurement. Health Place.

[18] Waverijn G, Wolfe MK, Mohnen S, Rijken M, Spreeuwenberg P, Groenewegen P (2014). A prospective analysis of the effect of neighbourhood and individual social capital on changes in self-rated health of people with chronic illness. BMC Public Health.

[19] Giordano GN, Mewes J, Miething A (2018). Trust and all-cause mortality: a multilevel study of US general social survey data (1978–2010). J Epidemiol Commun H.

[20] Glaeser EL, Laibson DI, Scheinkman JA, Soutter CL (2000). Measuring trust. Q J Econ.

[21] Putnam RD, Crothers L, Lockhart C (2000). Bowling alone: America’s declining social capital.

[22] Coleman JS (1988). Social capital in the creation of human capital. Am J Sociol.

[23] Maselko J, Hughes C, Cheney R (2011). Religious social capital: its measurement and utility in the study of the social determinants of health. Soc Sci Med.

[24] Dingemans E, van Ingen E (2015). Does religion breed trust? A cross-national study of the effects of religious involvement, religious faith, and religious context on social trust. J Sci Study Relig.

[25] Gabriel S, Valenti J, Naragon-Gainey K, Young AF (2017). The psychological importance of collective assembly: development and validation of the tendency for effervescent assembly measure (TEAM). Psychol Assess.

[26] McGuire MB (2008). Religion: the social context.

[27] Maselko J, Kubzansky LD (2006). Gender differences in religious practices, spiritual experiences and health: results from the US general social survey. Soc Sci Med.

[28] Maselko J, Kubzansky LD, Kawachi I, Staudenmayer J, Berkman L (2006). Religious service attendance and decline in pulmonary function in a high-functioning elderly cohort. Ann Behav Med.

[29] Hummer RA, Rogers RG, Nam CB, Ellison CG (1999). Religious involvement and US adult mortality. Demography.

[30] Wuthnow R (2002). Religious involvement and status-bridging social capital. J Sci Study Relig.

[31] Irwin J, LaGory M, Ritchey F, Fitzpatrick K (2008). Social assets and mental distress among the homeless: exploring the roles of social support and other forms of social capital on depression. Soc Sci Med.

[32] Stockdale SE, Wells KB, Tang L, Belin TR, Zhang L, Sherbourne CD (2007). The importance of social context: neighborhood stressors, stress-buffering mechanisms, and alcohol, drug, and mental health disorders. Soc Sci Med.

[33] Beyerlein K, Hipp JR (2005). Social capital, too much of a good thing? American religious traditions and community crime. Soc Forces.

[34] Blanchard TC, Bartkowski JP, Matthews TL, Kerley KR (2008). Faith, morality and mortality: the ecological impact of religion on population health. Soc Forces.

[35] Jylhä M (2009). What is self-rated health and why does it predict mortality? Towards a unified conceptual model. Soc Sci Med.

[36] Center R (2006). Social capital community survey: methodology and documentation.

[37] Sampson RJ, Raudenbush SW, Earls F (1997). Neighborhoods and violent crime: a multilevel study of collective efficacy. Science.

[38] RCMS:T2. All Religions. (Association of Statisticians of American Religious Bodies) In SocialExplorer.com*.* 2000. https://www.socialexplorer.com/tables/C2000/R11506925. Accessed 25 Aug 2017.

[39] Barber S, Hickson DA, Kawachi I, Subramanian SV, Earls F (2016). Neighborhood disadvantage and cumulative biological risk among a socioeconomically diverse sample of African American adults: an examination in the Jackson heart study. J Racial Ethn Health Disparities.

[40] Wodtke GT, Harding DJ, Elwert F (2011). Neighborhood effects in temporal perspective: the impact of long-term exposure to concentrated disadvantage on high school graduation. Am Sociol Rev.

[41] Huang FL (2016). Alternatives to multilevel modeling for the analysis of clustered data. J Exp Educ.

[42] Stroope S, Baker JO (2018). Whose moral community? Religiosity, secularity, and self-rated health across communal religious contexts. J Health Soc Behav.

[43] Murayama H, Fujiwara Y, Kawachi I (2012). Social capital and health: a review of prospective multilevel studies. J Epidemiol.

[44] Pinillos-Franco S, Kawachi I (2018). The relationship between social capital and self-rated health: a gendered analysis of 17 European countries. Soc Sci Med.

[45] Rupasingha A, Goetz SJ, Freshwater D (2006). The production of social capital in US counties. J Socio-Econ.

[46] Carpiano RM (2008). Actual or potential neighborhood resources and access to them: testing hypotheses of social capital for the health of female caregivers. Soc Sci Med.

[47] Putnam RD (1995). Tuning in, tuning out: the strange disappearance of social capital in America. PS: Pol Sci Politics.

[48] Mitchell CU, LaGory M (2002). Social capital and mental distress in an impoverished community. City Community.

[49] Liu B, Wright Austin SD, D’Andrá OB (2009). Church attendance, social capital, and black voting participation. Soc Sci Q.

[50] Campbell MK, Hudson MA, Resnicow K, Blakeney N, Paxton A, Baskin M (2007). Church-based health promotion interventions: evidence and lessons learned. Annu Rev Public Health.

[51] Villalonga-Olives E, Wind TR, Kawachi I (2018). Social capital interventions in public health: a systematic review. Soc Sci Med.

[52] Shiell A, Hawe P, Kavanagh S. Evidence suggests a need to rethink social capital and social capital interventions. Soc Sci Med. 2018. 10.1016/j.socscimed.2018.09.006. 10.1016/j.socscimed.2018.09.006 30219489

[53] Rudolph L, Caplan J, Ben-Moshe K, Dillon L. Health in All Policies: A Guide for State and Local Governments. Oakland: American Public Health Association and Public Health Institute; 2013.

